# Dynamic Analysis and Experimental Study of Lasso Transmission for Hand Rehabilitation Robot

**DOI:** 10.3390/mi14040858

**Published:** 2023-04-15

**Authors:** Jingxin Lu, Kai Guo, Hongbo Yang

**Affiliations:** 1 Suzhou Institute of Biomedical Engineering and Technology, Chinese Academy of Sciences, Suzhou 215163, China; 2 School of Mechanical and Electrical Engineering, Changchun University of Science and Technology, Changchun 130022, China; 3 School of Biomedical Engineering (Suzhou), Division of Life Sciences and Medicine, University of Science and Technology of China, Hefei 230026, China

**Keywords:** rehabilitation robot, lasso transmission, simulation

## Abstract

Lasso transmission is a method for realizing long-distance flexible transmission and lightweight robots. However, there are transmission characteristic losses of velocity, force, and displacement during the motion of lasso transmission. Therefore, the analysis of transmission characteristic losses of lasso transmission has become the focus of research. For this study, at first, we developed a new flexible hand rehabilitation robot with a lasso transmission method. Second, the theoretical analysis and simulation analysis of the dynamics of the lasso transmission in the flexible hand rehabilitation robot were carried out to calculate the force, velocity, and displacement losses of the lasso transmission. Finally, the mechanism and transmission models were established for experimental studies to measure the effects of different curvatures and speeds on the lasso transmission torque. The experimental data and image analysis results show torque loss in the process of lasso transmission and an increase in torque loss with the increase in the lasso curvature radius and transmission speed. The study of the lasso transmission characteristics is important for the design and control of hand functional rehabilitation robots, providing an important reference for the design of flexible rehabilitation robots and also guiding the research on the lasso regarding the compensation method for transmission losses.

## 1. Introduction

Stroke is a medical condition that can lead to the disability of middle-aged and elderly people. The seventh census found that China’s population is rapidly aging. The population aged 60 and above is 264 million, accounting for 18.70% of the total population, and the population aged 65 and above is 191 million, or 13.5% of the total population. Compared with the last decade, the increase rates for these two age groups are 2.51% and 2.72%, respectively. With the increase in the elderly population, stroke has become a common condition, and it often leads to motor dysfunction or even lifelong disability [1,2].

Stroke has the characteristics of a high incidence rate, disability rate, mortality rate, and recurrence rate. Most survivors of stroke experience the sequelae of hand dysfunction, and hand movement accounts for 80% of human upper limb movement, making it indispensable in daily life. Moreover, in recent years, the age of the incidence of stroke has decreased, and the rehabilitation training for the hand function of stroke survivors has become a major social problem to be solved [3,4,5]. A large number of patients demand scientific hand function rehabilitation equipment, and this demand has increased significantly because it can help patients carry out accurate, quantitative, and effective training. As a new field of robotics, over the past decade, rehabilitation robots have attracted more attention from researchers and rehabilitation doctors. Various hand rehabilitation robots have been developed to train the motor function of stroke patients’ hands [6,7,8,9,10]. Generally, hand rehabilitation robots can be divided into three categories: exoskeleton robots, flexible exoskeleton robots, and flexible gloves. The corresponding transmission modes are the connecting rod drive, rope drive, and pneumatic drive [11].

The transmission structure of an exoskeleton hand rehabilitation machine is generally rigid, and the linkage mechanism is usually used as the transmission structure to realize the underdrive design of fingers and to ensure the compactness of the structure and the number of motors in order to meet the requirements of lightweight design. It is easier to control than soft equipment. However, its limitation is that it is difficult to align with people’s finger joints and is uncomfortable and heavy [12,13,14,15,16]. For example, the lightweight under-actuated neurorehabilitation exoskeleton designed by Valladolid University, in which each finger is composed of an under-actuated connecting rod rotating mechanism, transmits the force of a linear actuator to the patient’s finger in the neurorehabilitation task [17]. Carbone et al. put forward a systematic design method for a new type of two-degree-of-freedom driving linkage mechanism used to assist in the movement of the finger exoskeleton. The finger exoskeleton has adaptability to the size of the finger, cost-effective design, and user-friendly function [18].

**Figure 1:**
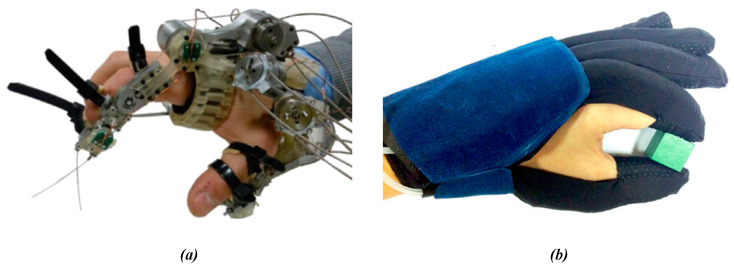
Flexible driving gloves. (**a**) HX-β; (**b**) Soft robot gloves.

Flexible exoskeleton hand function robots and flexible gloves mostly use rope transmission to provide power, and flexible gloves also have a way of transmitting force through compressed air [19,20,21]. Furthermore, the transmission driven by pneumatics and rope can reduce the weight and increase the compliance of the wearable robot system. By moving the remote host to a more convenient position, maintaining a rigid structure, or combined with a flexible fabric structure, this method frees the remote from the heavy driving unit and electronic equipment, thus increasing the portability, comfort, and usability of the equipment [22,23,24,25]. Although the development of this drive system aims to improve usability, reduce weight, and maximize compliance, the inevitable cost is the decrease in strength and accuracy compared with the traditional rigid exoskeleton [26,27,28,29]. As shown in Figure 1a, Dario Marconi designed a new index finger–thumb exoskeleton named HX-β for hand rehabilitation by lasso transmission, which allowed the thumb to flex/extend and rotate independently, thus realizing various natural and functional grasping configurations [30]. As shown in Figure 1b, Hong Kai Yap, National University of Singapore, designed a soft robot glove for hand function assistance of survivors after stroke and developed a new type of soft fiber fabric to strengthen the pneumatic actuator, which can reduce excessive action and provide better bending ability [31].

From the above analysis, it can be seen that structural design is important for the development of the hand-functional robot. The connecting rod structure is relatively thick and hard; therefore, it is difficult to realize multi-degree-of-freedom movement and maintain a large working space. Pneumatic and hydraulic transmission cannot be limited to plane motion, and its material is elastic and can be twisted, compressed, and wrinkled. This kind of motion can be regarded as providing an infinite number of degrees of freedom, which makes the control of the robot very challenging and requires new modeling, control, dynamics, and advanced planning methods. A lasso-driven rehabilitation hand is the most similar to the muscle drive of the human hand, which can realize movements similar to the human hand, easily produce higher fingertip force, and easily realize lightweight design [32,33]. This makes the hand rehabilitation robot based on lasso drive not bring burden to the hand during the rehabilitation process. bring burden to the hand during the rehabilitation process., which is more conducive to the long-term continuous training of patients. However, the production and maintenance of rope-driven rehabilitation gloves are complicated, resulting in increased costs. Even if a rope is broken or loose, it is difficult to repair it. Being lightweight and wearable are the needs of many patients. As one of the methods to realize the design of a lightweight hand robot, lasso transmission needs to be deeply analyzed in terms of its transmission structure design, the transmission characteristics of displacement and force in the transmission process, and the influence of different rope characteristics on transmission effect and service life to improve the performance of the rehabilitation robot.

## 2. Materials and Methods

### 2.1. Analysis of Physiological Structure and Rehabilitation Strategy of Hand

**Figure 2:**
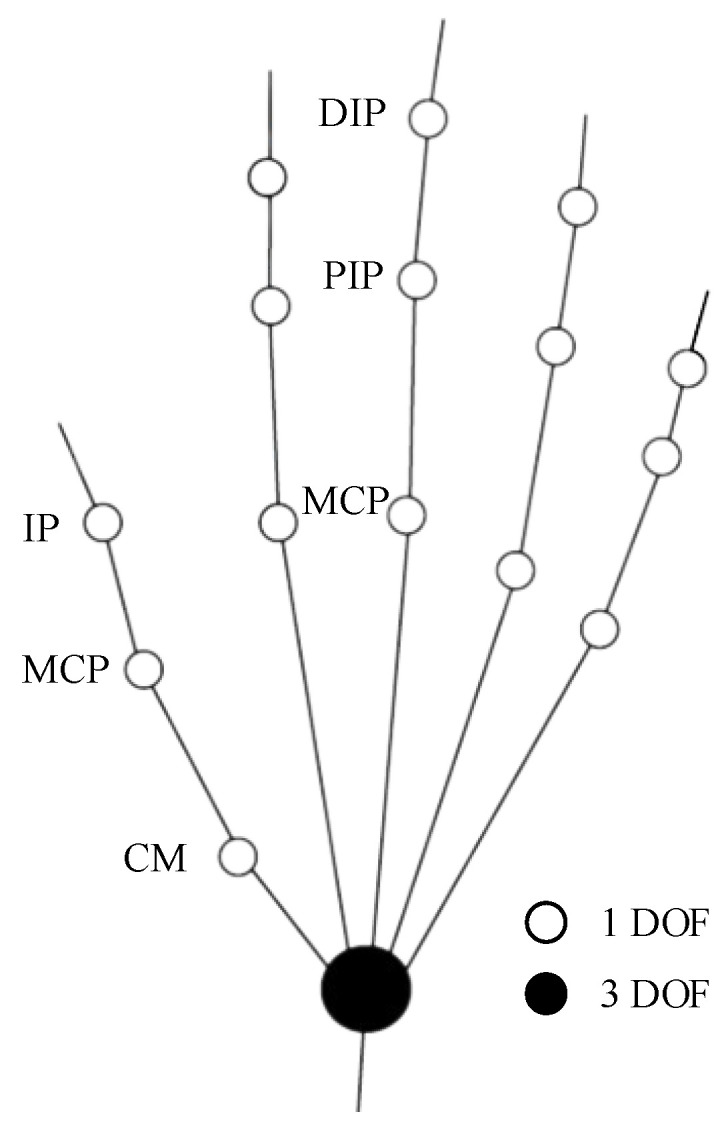
Schematic diagram of motion structure of hand connecting rod.

Each finger has multiple degrees of freedom, and each finger can be regarded as having three degrees of freedom after simplification. The fingers of a normal person’s hand are composed of four bones except the thumb, and the thumb is composed of three bones, which is one less bone than the other four fingers, but the metacarpals of the other four fingers are all fixed on the palm of the hand, and the degree of freedom is not increased because of the extra phalanges. Therefore, in the study of human hand movement, the thumb, like other fingers, can be considered as a joint structure with three degrees of freedom. As shown in Figure 2, in order to facilitate the design of hand functional rehabilitation robot, the motion model of fingers is simplified here, and all joints only consider the degrees of freedom of forward and backward bending. In this way, we obtain a schematic diagram of the motion structure of the hand linkage, and the white circle represents a degree of freedom (forward and backward bending). The black circle represents three degrees of freedom (bending back and forth, swinging left and right, and turning motion). For the thumb, IP, MCP, and CM in the figure respectively represent the pointed joint, metacarpophalangeal joint, and carpometacarpal joint of the thumb, while for other fingers, DIP, PIP, and MCP respectively represent the distal phalangeal joint, proximal phalangeal joint, and metacarpophalangeal joint of other four fingers.

Once the finger is disabled due to stroke or nerve injury, and if the patient has not used it for a long time, the patient’s hand behavior will be suppressed, the finger movement ability will be covered up, and inertia behavior will occur, which will lead to learned waste. In order to avoid this symptom and help the affected limb carry out rehabilitation training, continuous passive rehabilitation therapy was put forward. By assisting the patient’s fingers to carry out continuous passive exercise training, it can help the patient recover from the hand dysfunction as soon as possible. However, the number of traditional doctors is limited, and it is difficult for them to continue long-term and high-intensity rehabilitation training. The hand functional rehabilitation robot can solve this problem well. As long as the doctors set the rehabilitation mode, they can drive the patient’s fingers to carry out rehabilitation training.

### 2.2. Structure of Lasso in Hand Functional Rehabilitation Robot

**Figure 3:**
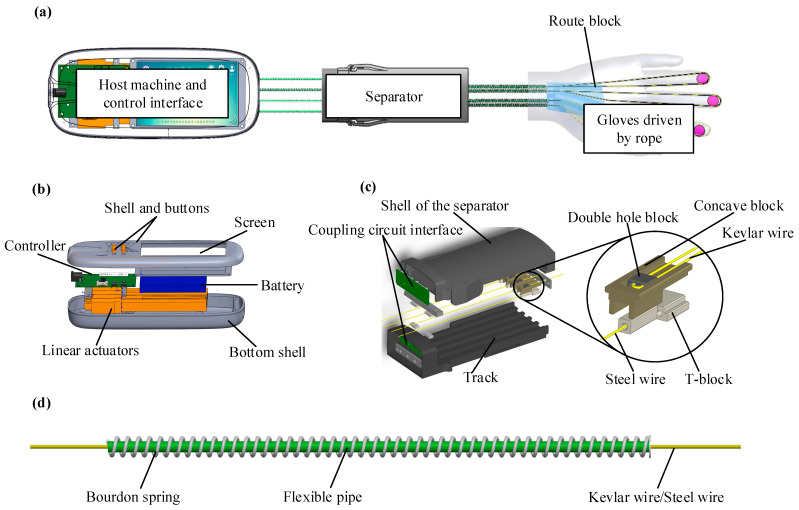
Structure diagram of hand functional rehabilitation robot. (**a**) Overall structure diagram of hand rehabilitation robot [34,35,36]; (**b**) Control host; (**c**) Separation transmission platform; (**d**) Lasso transmission structure.

The overall structure of the hand rehabilitation robot is shown in Figure 3a. Figure 3b shows the control host, Figure 3c shows the separation transmission platform, and Figure 3d shows the lasso transmission structure. Wearable gloves are made of lightweight and delicate microfiber cloth. Pressure sensors are arranged at the fingertips (connected to the control board along with the transmission mechanism). Kevlar wires that provide torque for finger bending pass through the path block in the palm of the hand and are connected to the double-hole block through the hose in the spring. The double-hole block is fixed on the concave block to establish a matching connection relationship with the T-shaped block. The T-block is fixed with the steel wire rope, which is connected to the motor slider through the hose in the spring. When the motor rotates, the slider drives the steel wire rope to move, the steel wire rope drives the T-shaped block to move linearly on the track, and the Kevlar wire is tensioned to drive the fingers to bend.

As shown in Figure 3b, the host of the whole equipment is mainly composed of the control board, motor, battery, and screen. Besides controlling the movement position of the motor, the control board is also responsible for monitoring the pressure value collected by the fingertip pressure sensor. In the case of insufficient fingertip force, the power-assisted mode can be selected to provide grasping force for the fingers. The pressure sensor for monitoring fingertip force is first connected to the coupling circuit board, both of which are then connected to the control host with the steel wire spring tube, and then finally connected to the control board.

As shown in Figure 3c, the separation transmission platform is mainly composed of a shell with rails, a T/concave block, and a coupling circuit board. The main purpose of the separation design is to realize the applicability of the host, and the same host can be used for the motion control requirements of the left and right hands. At the same time, it is convenient for the separation structure to be sent to different places for processing. Here, the two ends of the spring tube are fixed to the main machine and the separation transmission mechanism, and the separation transmission mechanism and the palm path block are also connected through the spring tube. To reduce friction, the air pipe is installed in the path block, and the hose is installed in the spring pipe to enhance the transmission effect and service life.

### 2.3. Simulation and Transmission Characteristics Analysis of Lasso

The structure and parameters of the lasso affect its performance, but there is still a lack of relevant research. In this study, through the process of structural modeling, simulation, and mathematical modeling of the lasso, the influence of the parameters of the lasso on its performance is studied.

#### 2.3.1. Transmission Characteristics of Lasso Micro-Element

**Figure 4:**
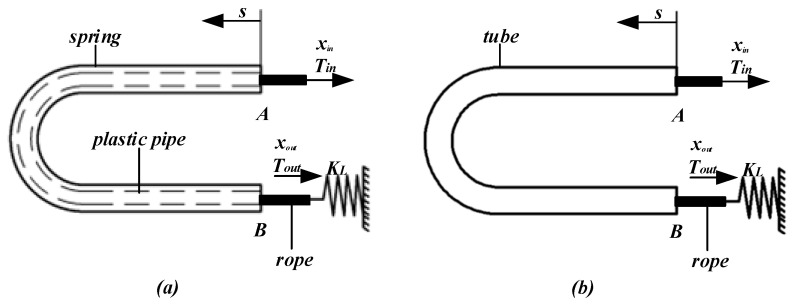
(**a**) Spatial arbitrary rope model diagram; (**b**) Simplified model of lasso system.

Figure 4a shows an arbitrary lasso model in space; the lasso structure consists of springs, plastic pipes, and ropes. For the convenience of modeling, the plastic pipes and springs are simplified into a complete set of pipes, as shown in Figure 4b, and the friction factor is adjusted for analysis. The input force ($T_{\mathrm{in}}$) or displacement ($x_{\mathrm{out}}$) of the rope is provided by the power drive mechanism, the output end is connected to the load with the elastic coefficient ($k_{L}$), end A and end B of the sleeve are relatively fixed, and s refers to the point with the arc length s along the rope direction from point A.

The initial pre-tightening force of the rope is zero, and given any positive displacement of the rope, the end of the rope responds to the output. κ(s) and τ(s) indicate the bending and twisting degree of the lasso, respectively. Because the unit mass of the lasso is small, the friction between lassos is mainly caused by the external tension of the bent rope; therefore, the static model of arbitrary curve lasso transmission in space can be established by the micro-element analysis method.

**Figure 5:**
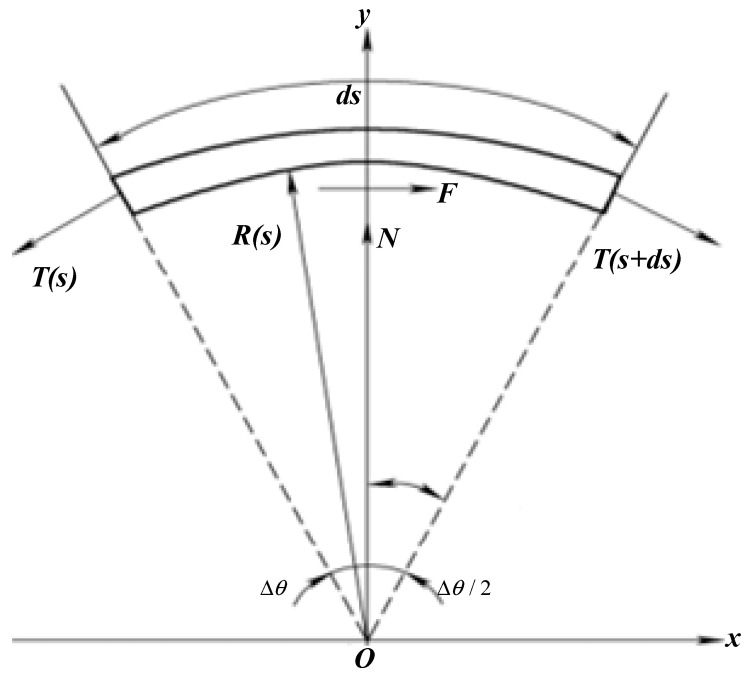
Static analysis diagram of rope micro-element transmission.

The rope elements are so small that each segment is a plane curve, as shown in Figure 5, and the mass is negligible. The twist angle around the axis of the rope between the close planes of adjacent elements does not affect the transmission of axial tension between two elements, that is, the output tension of the ith segment element is equal to the input tension of the (i+1)th segment element. Therefore, the transmission characteristics of lasso force and displacement can be studied by analyzing the axial tension transmission of microelements.

Here, we have established an ideal transmission model. Assuming that there are no factors, such as backlash, collisions, and deformation, we can build a simplified model only considering the influence of friction factors in the transmission process. As shown in Figure 5, the arc length of the rope microelement at a point S is ds, T(s) and T(s+ds) are the tensile forces on both ends of the microelement, N and F are the normal pressure and friction between the casing and the rope, respectively, R is the curvature radius of the microelement, and $\dot{s}$ is the movement speed of the rope microelement relative to the casing. The force balance equation is as follows:

(1) $$\{\begin{array}{l} F+T(s+ds)\cos\frac{\Delta \theta }{2}-T(s)\cos\frac{\Delta \theta }{2}=0\\ T(s)\sin\frac{\Delta \theta }{2}+T(s+ds)\sin\frac{\Delta \theta }{2}-N=0 \end{array}$$

where Δθ=ds/R=κ(s)ds, $F=\mu N\mathrm{sgn}(\dot{s})$, and μ is the friction factor.

When $\dot{s}\neq 0$, and the rope element is moving, the output force T(s) and deformation δ(s) of the element can be expressed as:

(2) $$\frac{d\Omega }{ds}=B\Omega$$

where $\Omega ={\left(\begin{array}{ll} T(s) & \delta (s) \end{array}\right)}^{T}$ and $B=\left(\begin{array}{cc} -\mu \kappa (s)\mathrm{sgn}(\dot{s}) & 0\\ 1/EA & 0 \end{array}\right)$, where E is the element elastic modulus, and A is the cell cross-sectional area.

From the analysis of the rope micro-element in the moving state, the relationship between the rope force and displacement output at point s, where the micro-element is located, can be obtained as follows:

(3) $$\{\begin{array}{l} T(s)=T_{\mathrm{in}}\exp[-\mu \theta (s)\mathrm{sgn}(\dot{s})]\\ x(s)=x_{\mathrm{in}}-\delta (s) \end{array}\;0\leq s\leq L_{1}$$

where $\delta (s)=T_{\mathrm{in}}L_{\mu \theta (s)}/EA$, $\theta (s)=\int_{0}^{s}\kappa (s)ds$, and $L_{\mu \theta (s)}=\int_{0}^{s}\exp[-\mu \theta (s)\mathrm{sgn}(\dot{s})]ds$.

When the whole rope moves, all the micro-elements move in the same direction. When the elongation of the rope increases ($\dot{s}>0$), the rope is in the stretching range. Conversely, when the elongation of the rope decreases ($\dot{s}<0$), it is in the relaxation zone. At this time, the relationship between the lasso force and the displacement output is as follows:

(4) $$\{\begin{array}{l} T_{\mathrm{out}}=T_{\mathrm{in}}\exp[-\mu \theta (L)\mathrm{sgn}(\dot{s})]\\ x_{\mathrm{out}}=x_{\mathrm{in}}-\delta (L) \end{array}$$

The end of the lasso transmission system is an elastic load. Using Hooke’s law for the load, we can obtain the relationship between the output force and the input displacement of the rope end as follows:

(5) $$T_{\mathrm{out}}=k_{t}x_{\mathrm{in}}$$

where $\frac{1}{k_{t}}=\frac{1}{k_{e}}+\frac{1}{k_{L}}$ and $k_{e}=\frac{EA}{\exp[-\mu \theta (L)\mathrm{sgn}(\dot{s})]L_{\mu \theta (L)}}$, where $k_{t}$ is the equivalent elastic coefficient of the lasso transmission system, $k_{e}$ is the rope equivalent elastic coefficient, and $k_{L}$ is the load elasticity coefficient.

It can be seen from Formula (5) that the spatially arbitrary curvilinear lasso transmission system can be regarded as a series connection of two springs with elastic coefficients $k_{e}$ and $k_{L}$. $k_{e}$ is related to the elastic modulus of the rope, the cross-sectional area, the friction coefficient between the contact surfaces of the lasso, and the full curvature, and it determines the relationship between the input displacement and the output force.

#### 2.3.2. Motion Simulation of Lasso Model

We used the LS-DYNA module in ANSYS Workbench to simulate the dynamic process of lasso transmission, and the results are shown in Figure 5. The transmission part of the spring lasso includes a bourdon spring, flexible tube, and steel wire, which were imported into Workbench for grid division.

**Figure 6:**
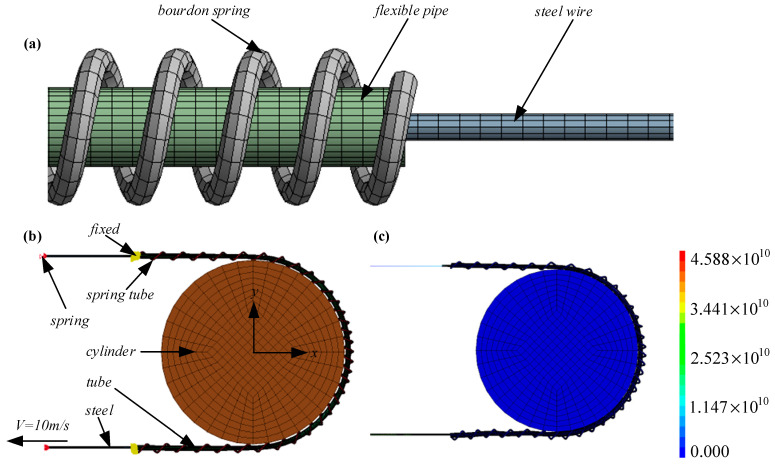
Lasso simulation modeling analysis diagram. (**a**) Grid division diagram; (**b**) K file model; (**c**) Dynamic equivalent stress cloud chart.

As shown in Figure 6a, the spring adopted the sweep grid division method, and the rest adopted the multi-area grid division method, which divided 95,615 elements and 140,680 nodes. After grid division, it was imported into LS-PrePost for model processing. First, a cylinder with a radius of 50 mm was established, and each PART was given material properties. To simplify the simulation process, Q235 material was used for the simplified treatment of the spring and tube, the steel wire was made of elastomer material, and the cylinder had a rigid body. The motion of the cylinder’s rigid body was defined to make contact with the lasso structure, and the deformed K file model was generated, as shown in Figure 6b. This model was edited, the two ends of the tube and the spring were constrained, the cylindrical rigid body was then fixed, the dynamic friction coefficient was set to 0.2, and the contact stiffness coefficient was set to 2. A load spring was established; the elastic stiffness was 100,000 N/m, and a uniform load of 10 m/s was applied at one end of the steel wire. The result of solving the dynamic equivalent stress cloud chart is shown in Figure 6c.

### 2.4. Lasso Transmission Experiment

**Figure 7:**
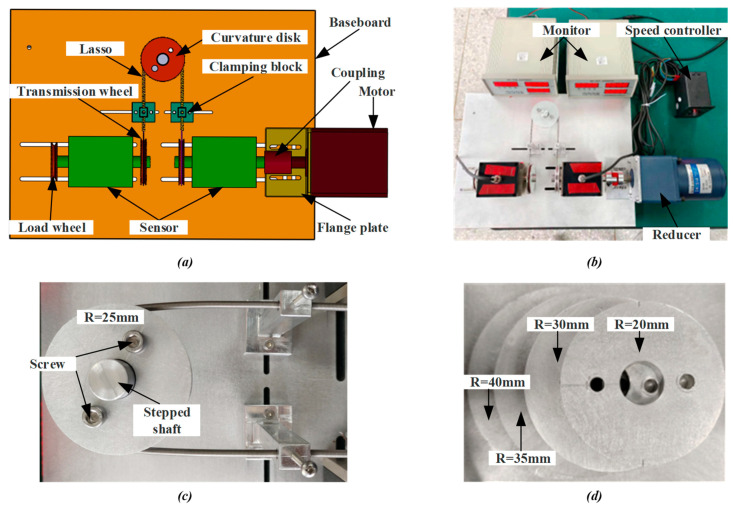
Lasso transmission characteristic analysis test platform. (**a**) Model diagram of test platform; (**b**) Physical diagram of test platform; (**c**) Assembly drawing of lasso mechanism; (**d**) Disc with different curvatures.

To determine the relationship between the input and output characteristics of lasso transmission, we set up a test platform for actual measurement and then observed and recorded the effects of different speeds and total curvatures on the rope transmission effect. The test bed was mainly composed of a speed-regulating motor (rotating speed range 7–14 r/min), a reducer, coupling, a dynamic torque sensor, a lasso transmission system, and load weight. The motor provided different speeds for the transmission experiment, weights were loaded on the load wheel to ensure a constant load, and the monitor recorded the real-time values of the dynamic torque and speed of the lasso input and output. The three-dimensional model diagram and the physical diagram of the lasso transmission characteristic analysis test bed are shown in Figure 7a,b, respectively. The disks with a radius of R = 20, 25, 30, 35, and 40 mm (as shown in Figure 7d) were fixed on the stepped shaft, providing different curvatures for the lasso. The assembly of the lasso mechanism is shown in Figure 7c.

## 3. Result and Discussion

### 3.1. Simulation Result of Force, Velocity and Displacement of Lasso

**Figure 8:**
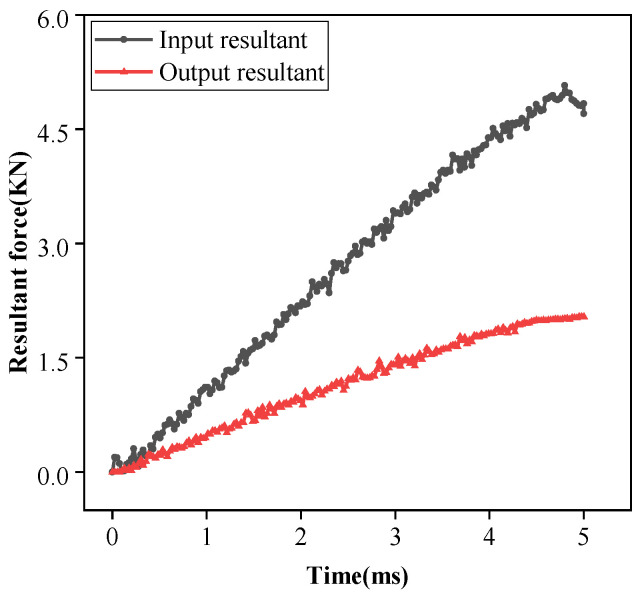
The resultant force of steel input and output varies with time.

From the results of the dynamic simulation analysis, the data of the resultant force of steel wire input and output with time were extracted, and Figure 8 was created after data processing. It can be seen that under all the above conditions, the curve of the input force and output force at both ends of the steel wire tends to be a straight line with a certain slope in 5 ms. At 4.5 ms, the resultant force difference is 2.784 KN. From Figure 8, it can be seen that the elongation of the spring is 19.9 mm, resulting in a tensile force of 1.99 KN, and the remaining resultant force difference of 0.794 KN comes from the friction between lassos. Because the elastic force of the load spring is linear, it can be speculated that the friction force also increases linearly.

**Figure 9:**
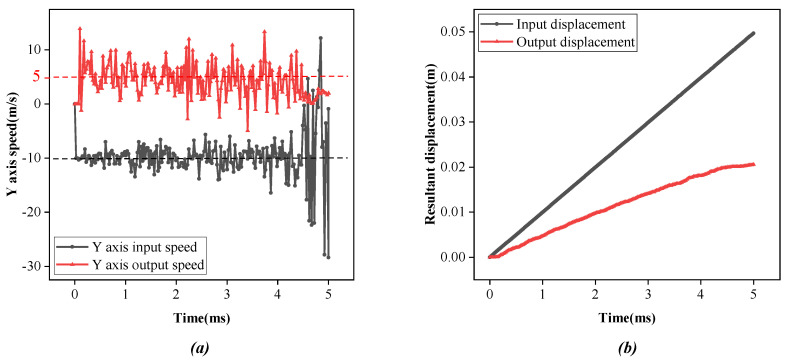
Simulation analysis curve of lasso movement speed and displacement. (**a**) Speed-time curve; (**b**) Displacement-time curve.

From the simulation results, the data of *Y*-axis displacement and speed of steel wire input and output with time were extracted, and Figure 9 was created after data processing. The speed-time curve in Figure 9a shows that over 5 ms, the input speed of steel is about −10 m/s, the output speed is about 5 m/s, and the speed of the steel wire is about 5 m/s lower after being driven by lasso. The displacement-time curve in Figure 9b shows that, in the same time duration, the *Y*-axis displacement of steel at the input end is 49.7 mm, and the *Y*-axis displacement of steel at the output end is 20.6 mm. Steel loses about 39.1 mm in displacement after lasso transmission. The measurement of the element divided into grids shows that each element stretches about 0.025 mm, indicating that the loss of speed and displacement of steel from the input end to the output end can be attributed to the elastic deformation of the elastomer, and the rest reflects the deformation of the lasso.

### 3.2. Result of Experiment

**Figure 10:**
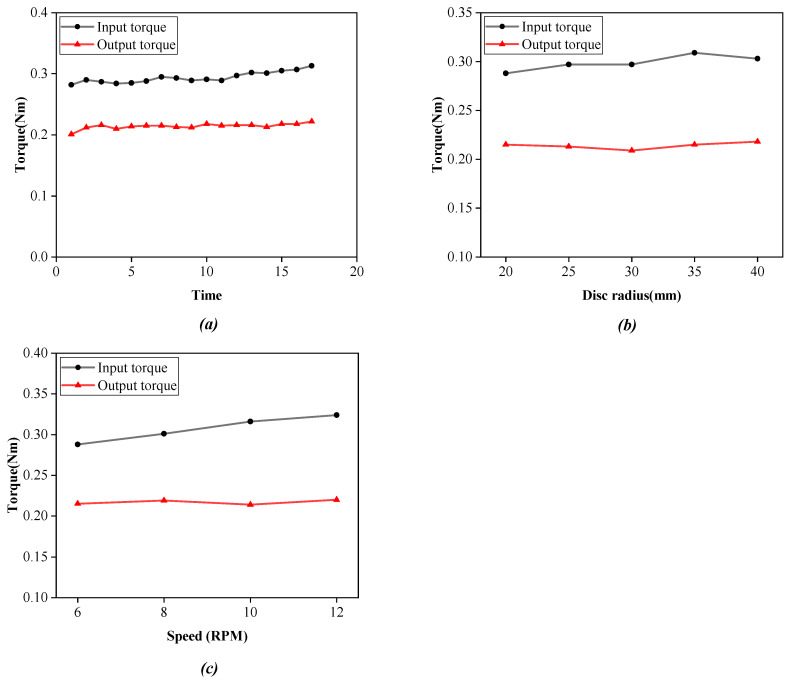
Experimental data curve of lasso input and output characteristics. (**a**) Data diagram of lasso input and output torque; (**b**) Torque-curvature radius diagram; (**c**) Torque-speed diagram.

In this paper, the lasso system test platform we built was used to measure the input and output characteristic data of the lasso. First, under the conditions of a curvature radius of R = 20 mm, a load of 1 kg, and a speed of 6 rpm, and the data of 17 sets of lasso transmission input and output torques were recorded, as shown in Figure 10a. It can be observed that the output torque is smaller than the input torque, and the average value shows that there is a torque loss of 0.08 Nm. Second, under the condition of a load of 1 kg and a speed of 6 rpm, the data of input and output torques of the lasso transmission with different curvature radii are shown in Figure 10b. It can be observed from the figure that with the increase in the lasso curvature, the loss between input torque and output torque of the lasso transmission also increases. Finally, under the condition of a curvature radius of R = 20 mm and a load of 1 kg, the data of input and output torques of the lasso transmission at different speeds are shown in Figure 10c. It can be observed that with the increase in speed, the loss between input torque and output torque of the lasso transmission also increases.

From the above experimental data and image analysis, it can be said that there is torque loss in the process of lasso transmission, and with the increase in the lasso curvature radius and transmission speed, the torque loss increases correspondingly. This provides a theoretical reference for us to design a lasso drive robot.

## 4. Physical Prototype Experiment of Robot

The actuator of the hand functional rehabilitation robot is a glove, and the whole flexible actuator looks like a glove. However, flexible electronics products, sensors, and ropes are embedded in the glove to help the hand functional rehabilitation robot control the bending angle of fingers through the ropes. Therefore, the workspace of the rehabilitation robot is consistent with the human hand.

**Figure 11:**
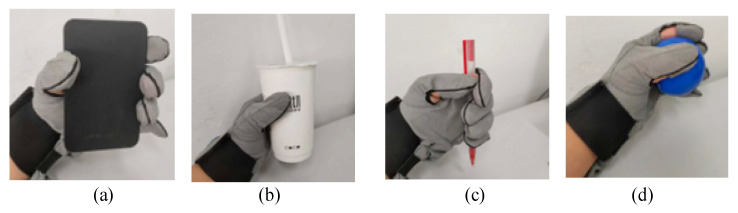
Grasping training effect. (**a**) Finger grasping square box; (**b**) Finger grasping milk tea; (**c**) Finger grasping pen; (**d**) Finger grasping the ball.

For patients with hand dysfunction, grasping ability is one of the abilities they need most, because grasping exercise is the most commonly used action in daily life. Patients’ illness will lead to muscle weakness or stiffness in their hands, which makes it difficult for them to complete this action, resulting in anxiety-related psychological problems, which are unfavorable for the recovery of finger function. The primary function of the hand function robot we designed is to help the patient complete the grasping exercise training. As shown in Figure 11, after wearing the hand function rehabilitation robot, we began to grasp the object, grasping the hard disk box, milk tea, writing pen, and balloon, respectively, and the grasping effect is shown in Figure 11. It can be observed that the hand functional rehabilitation robot performs well in the user’s grasping action and can adaptively match objects with different shapes and sizes, and the contact between fingers and objects has a certain pressure, which indicates that the hand functional rehabilitation robot has the function of satisfying the grasping training of patients.

**Figure 12:**
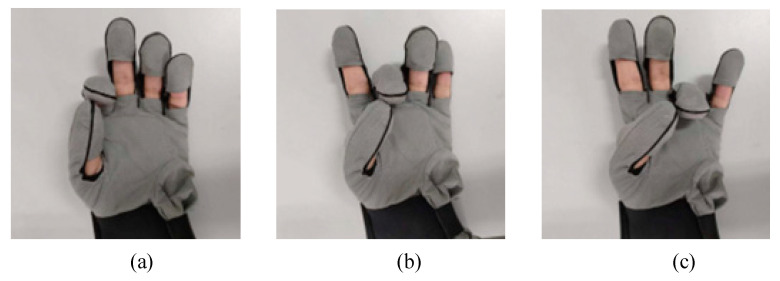
Finger training effect. (**a**) Index finger training; (**b**) Mid-finger training; (**c**) The ring finger training.

In addition to grasping experiment, we have also experimented with finger movement training, for which we set up three finger-pointing training modes. More diverse training methods are helpful to the recovery of patients. As shown in Figure 12, the finger-pointing training modes of the index finger, middle finger, and ring finger are carried out, respectively. From the finger-pointing training diagram, it can be seen that the action of the actuator in helping the finger to move is close to accuracy.

**Figure 13:**
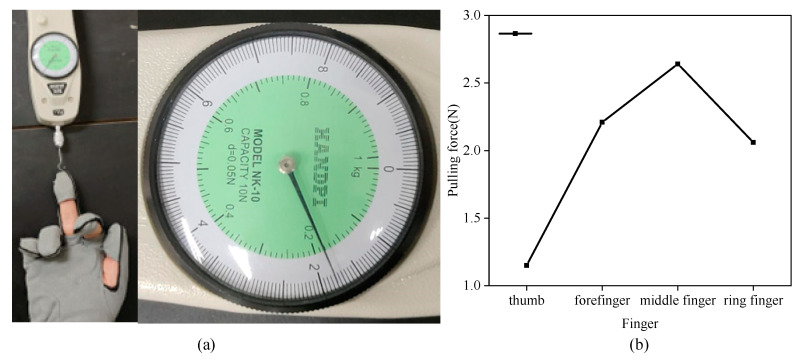
Finger pull test in rehabilitation training mode. (**a**) Finger tension measurement experiment; (**b**) Average diagram of tension on each finger.

When the hand functional rehabilitation robot drives the fingers to train, we collect the pulling force data provided by the hand functional rehabilitation robot for the fingers. As shown in Figure 13a, the finger pulling force is measured by the tensioners, and the fingers and the tensioners are fixed to ensure that the initial value of the pulling force is zero, and the fingers are always in a naturally relaxed state. After the rehabilitation training begins, the tension data is recorded manually by the tension device, and each finger collects five groups of tension data, and the stress diagram of each finger in the training mode of the rehabilitation robot is drawn by taking the average value of the data. As shown in Figure 13b, the pulling force provided by the robot for the finger ranges from 1 N to 3 N, and the longer the finger is, the greater the deformation generated in the same motor pulling displacement and the greater the pulling force received by the finger. We can adjust the stroke of the motor to control the deformation of the flexible gloves, and then change the pulling force provided by the robot for each finger. The stroke of the motor is limited by the structure, which also ensures the safety of the fingers.

Based on the above contents, we can draw a conclusion. From the experiment of rehabilitation training mode of hand functional rehabilitation robot, we can see that the robot can drive fingers to carry out rehabilitation training and provide patients with the help needed to meet the hand movement requirements in their daily life.

## 5. Discussion and Conclusions

In this paper, based on the structural design of hand functional rehabilitation robot driven by a lasso, the influence of the movement speed and total curvature of the lasso on the transmission characteristics of the lasso was studied. By analyzing the transmission characteristics of the lasso, the research provided a compensation basis and guidance for the movement control of the lasso system, reduced the kinematic loss of the lasso in the transmission process, made the lasso transmission system more time-effective, and improved the accuracy of the movement speed and transmission torque. There has been a lack of research on lasso transmission characteristics with different loads, material properties, and friction coefficients in the experiment. Because of the great difference in loads under different working conditions, it is necessary to analyze the specific conditions. Therefore, future research will focus on the influence of different material properties and transmission system friction on transmission characteristics to obtain a more reliable lasso transmission system.

This paper focused on the sequelae of hand dysfunction in stroke survivors and designed the structure of a hand rehabilitation robot. The transmission mode is flexible lasso transmission, and the static micro-element analysis of the lasso transmission model was carried out. The results indicate that the input and output characteristics of an arbitrary curve lasso transmission system in space are related to the elastic modulus of the rope, cross-sectional area, and friction coefficient between the lasso contact surfaces and the total curvature.

The lasso transmission model was established, and the dynamic simulation analysis and data acquisition for the test were carried out. The dynamic solution results and experimental data indicate that the lasso system can meet the speed and force required by the hand rehabilitation robot as a transmission mechanism, but there were response losses of force, speed, and displacement in the lasso transmission of steel, and the reasons for the loss can be analyzed, which provided a basis for the optimization of the lasso transmission system of the hand functional rehabilitation robot.

After the design and manufacture, the hand functional rehabilitation robot was tested by patients, and the rehabilitation experiments of finger grasping training and finger training mode were carried out respectively. The experimental results show that the hand functional rehabilitation robot based on lasso transmission can help fingers to carry out continuous and passive hand functional rehabilitation training.

## Data Availability

The data presented in this study are available on request from the corresponding authors.
